# A study on the impact of chronic diseases and depressive symptoms comorbidity on the risk of cognitive impairment in middle-aged and older adults people based on the CHARLS database

**DOI:** 10.3389/fpubh.2025.1558430

**Published:** 2025-02-27

**Authors:** Biqi Zu, Ning Wang, Lijun Fan, Jinsong Huang, Yunan Zhang, Yulan Wu, Maosheng Du

**Affiliations:** ^1^Psychiatry Department, Dalian No.7 People’s Hospital, Dalian, China; ^2^Obstetrics Department, Dalian Women and Children’s Medical Center (Group), Dalian, China

**Keywords:** chronic diseases, depressive symptoms, cognitive impairment, overlap weighting, mental health

## Abstract

**Objective:**

This study aimed to investigate the combined impact of comorbid chronic diseases and depressive symptoms on the risk of cognitive impairment in middle-aged and older adults populations. It also explored the interaction mechanisms and provided scientific evidence for cognitive health interventions.

**Methods:**

Data from the 2020 wave of the China Health and Retirement Longitudinal Study (CHARLS) were used, including 16,890 participants aged 45 years and older. Overlap weighting was applied to control for confounding factors such as gender, age, BMI, ADL, and smoking status. Multivariate logistic regression models assessed the combined effect of chronic diseases and depressive symptoms on cognitive impairment risk. Subgroup analyses were conducted to explore gender, age, and education level differences. Sensitivity analyses, including propensity score matching (PSM) and E-value estimation, were performed to evaluate the robustness of the findings.

**Results:**

The coexistence of chronic diseases and depressive symptoms significantly increased the risk of cognitive impairment, with an adjusted odds ratio (OR) of 1.22 (95% CI: 1.14–1.31, *p* < 0.05). Subgroup analyses revealed that this combined effect was more pronounced in males, individuals aged ≥60 years, and those with lower education levels (elementary school or below). Overlap weighting effectively balanced baseline characteristics, while sensitivity analyses and E-value calculations confirmed the robustness of the results.

**Conclusion:**

Comorbid chronic diseases and depressive symptoms exert a significant cumulative effect on cognitive impairment risk in middle-aged and older adults populations through complex interaction mechanisms. This study addresses a research gap and provides evidence for personalized disease management and psychological interventions. Future research should further explore the mechanisms of these interactions and validate the findings in diverse populations to enhance generalizability.

## 1 Introduction

With the acceleration of global population aging, cognitive impairment among middle-aged and older adults populations has gradually become a focal point in both societal and medical research. Cognitive impairment not only significantly impacts individuals’ daily lives and social functioning (1, 2), but it also greatly increases the healthcare burden on families and society (3), and in severe cases, can lead to more serious health problems, such as dementia (4). It is estimated that there were approximately 962 million people aged 60 and above in 2017, with projections ranging from 1.4 billion to 2.1 billion by 2050 (5), and the incidence of cognitive impairment and dementia increases significantly with age, which has sparked widespread concern. Against this backdrop, more research has started focusing on the risk factors for cognitive impairment, especially in the context of chronic diseases and depressive symptoms (6).

Chronic diseases are common health issues among middle-aged and older adults populations, including hypertension, diabetes, and coronary heart disease. The relationship between these chronic diseases and cognitive impairment has been confirmed in numerous epidemiological studies (7, 8). Chronic diseases may increase the risk of cognitive impairment through mechanisms that affect brain structure and function, such as vascular damage (9), metabolic disorders (10), and inflammatory responses (11). At the same time, depressive symptoms are also prevalent psychological health issues in the older adults, and they are recognized as independent risk factors for cognitive impairment. Depressive symptoms may directly damage brain function through prolonged stress and elevated cortisol levels (12), and they may also reduce social activity and cognitive engagement, further accelerating cognitive decline (13).

Although many studies have explored the independent effects of chronic diseases and depressive symptoms on cognitive impairment (14–19), chronic diseases and depressive symptoms are closely related and often coexist in individuals (20, 21). However, the combined impact of their comorbidity on cognitive impairment has been less studied. Most existing research focuses on the individual effects of chronic diseases or depressive symptoms on cognitive function, overlooking how these two factors might interact to jointly exacerbate the risk of cognitive impairment. Therefore, exploring the effect of the comorbidity of chronic diseases and depressive symptoms on cognitive impairment and their potential interaction mechanisms has become an important research topic.

Based on the limitations of existing research, this study aims to explore how the comorbidity of chronic diseases and depressive symptoms jointly affects the risk of cognitive impairment in middle-aged and older adults populations. Specifically, we hypothesize that the comorbidity of chronic diseases and depressive symptoms may jointly exacerbate the occurrence of cognitive impairment through various complex biological and psychological mechanisms. Chronic diseases may affect brain function through physiological mechanisms, such as vascular damage, inflammation, and metabolic abnormalities (9, 10), while depressive symptoms may further promote cognitive decline through prolonged stress, social isolation, and reduced cognitive engagement (11, 12). We propose the following research hypothesis: the comorbidity of chronic diseases and depressive symptoms will significantly increase the risk of cognitive impairment in middle-aged and older adults individuals, and they may interact through biological and psychological mechanisms to accelerate cognitive decline.

Although chronic diseases and depressive symptoms have both been widely studied and are closely associated with the occurrence of cognitive impairment, research on their combined effects on cognitive impairment is still relatively scarce. Existing literature mainly focuses on the individual mechanisms of chronic diseases and depressive symptoms on cognitive function, overlooking their potential interaction. Particularly in the older adults population, the coexistence of chronic diseases and depressive symptoms is very common (22, 23), but how these comorbid conditions affect the risk of cognitive impairment and their potential interaction mechanisms have not been deeply explored. Filling this gap will help provide a comprehensive understanding of the complex mechanisms underlying cognitive impairment and offer more precise theoretical support for health interventions targeting the older adults population.

This study aims to systematically analyze the combined impact of comorbid chronic diseases and depressive symptoms on the risk of cognitive impairment in middle-aged and older adults individuals. By exploring the interaction effects of chronic diseases and depressive symptoms in this group, we will further reveal how these factors jointly exacerbate cognitive decline through biological mechanisms (e.g., vascular damage, inflammation) and psychological mechanisms (e.g., stress, reduced social activity). The novelty of this research lies in its emphasis on the interaction between these two factors in the comorbid state, filling a research gap regarding the combined effects of chronic diseases and depressive symptoms on cognitive impairment. This study aims to provide new scientific evidence for cognitive health interventions in the older adults, promoting the development of personalized disease management and psychological intervention strategies. Specifically, this research will offer more precise and effective intervention recommendations for public health policy and cognitive health in an aging society.

## 2 Materials and methods

### 2.1 Materials source

The China Health and Retirement Longitudinal Study (CHARLS) is a longitudinal survey of the health and retirement status of a nationally representative population aged 45 years and older in mainland China. This survey collected data from 2011 to 2020, making it the first nationally representative survey of middle-aged and older adults populations in China. CHARLS provides a high-quality, nationwide public micro-database.

This study utilized data from the 2020 wave (Wave 5) of CHARLS. Ethical approval for the study was obtained from the Biomedical Ethics Committee of Peking University (Approval Number: IRB00001052-110155). During the survey, all participants who consented to participate signed an informed consent form.

### 2.2 Inclusion and exclusion criteria

**Figure 1:**
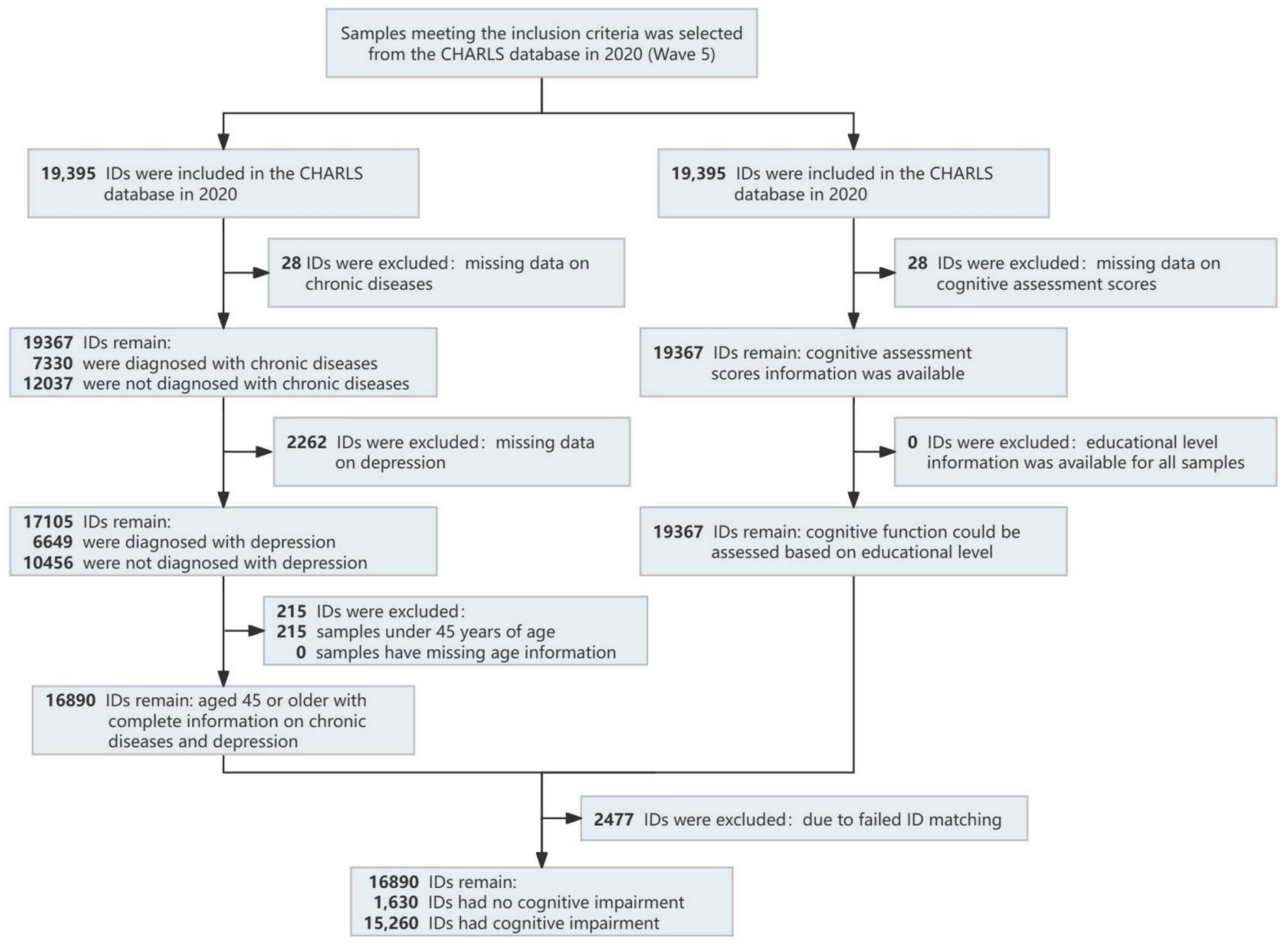
Data cleaning flowchart.

Inclusion criteria: ① Complete data available for chronic disease diagnoses. ② Complete data available for depressive symptom assessment. ③ Complete data available for cognitive function scores. ④ Age ≥ 45 years. Exclusion Criteria: Missing information on educational attainment. The data cleaning process is shown in Figure 1. In this study, IDs with both chronic diseases and depressive symptoms are categorized as the case group, while IDs without the simultaneous presence of chronic diseases and depressive symptoms are categorized as the reference group. The analysis focuses on the impact of the comorbidity of chronic diseases and depressive symptoms on cognitive function.

### 2.3 Chronic disease assessment

The assessment of chronic diseases was based on responses to the CHARLS questionnaire item: “Has a doctor ever diagnosed you with any of the following chronic diseases?” The chronic diseases included in this study were hypertension, dyslipidemia (including hyperlipidemia or hypolipidemia), diabetes or elevated blood glucose (including impaired glucose tolerance or fasting hyperglycemia), cancer or other malignant tumors (excluding mild skin cancer), chronic lung diseases (e.g., chronic bronchitis or emphysema), pulmonary heart disease (excluding tumors or cancers), liver diseases (excluding fatty liver, tumors, or cancers), heart diseases (e.g., myocardial infarction, coronary heart disease, angina, congestive heart failure, and other heart conditions), stroke, kidney diseases (excluding tumors or cancers), stomach or digestive system diseases (excluding tumors or cancers), emotional or mental health issues, memory-related diseases (e.g., Alzheimer’s disease, brain atrophy), Parkinson’s disease, arthritis or rheumatism, and asthma (not related to lung diseases), totaling 15 types of chronic diseases. If an individual self-reports having one or more chronic diseases in the survey, they are considered to have chronic diseases. If the individual does not report any chronic diseases, they are considered not to have chronic diseases.

### 2.4 Depression assessment

Depression symptoms were screened using the 10-item Center for Epidemiological Studies Depression Scale (CESD-10) (24) based on participants’ status over the past week (feelings and behaviors from the previous week) in the CHARLS questionnaire. The scale includes 10 items (eight negative and two positive). The negative items include: “I worry about little things,” “I have trouble concentrating while doing things,” “I feel sad,” “I feel that everything takes effort,” “I feel afraid,” “My sleep is restless,” “I feel lonely,” and “I feel I cannot go on with my life.” The positive items include: “I have hope for the future” and “I feel good.” Each item has four response options: “rarely or none of the time,” “not very much,” “some of the time or about half the time,” and “most of the time,” which are assigned scores from 0 to 3 (the positive items are reverse scored). The total score ranges from 0 to 30, with higher scores indicating more severe depression symptoms. In this study, depression symptom screening is classified into negative and positive categories, with scores below 10 considered no depressive symptoms, and scores of 10 or above considered positive for depression symptoms. The CESD-10 has demonstrated good reliability and validity, with a Cronbach’s α of 0.78, effectively screening for depression symptoms in the older adults population (25).

### 2.5 Cognitive function assessment

Cognitive function in this study was assessed using the Mini-Mental State Examination (MMSE) (26), a widely recognized tool for evaluating cognitive impairment and intellectual functioning. The MMSE provides a comprehensive and accurate reflection of the degree of cognitive deficits and intellectual levels, serving as a basis for neuropsychological diagnosis and treatment. The MMSE covers multiple cognitive domains, including memory, orientation, comprehension, attention, and reading/constructional abilities. The total score ranges from 0 to 30, with higher scores indicating better cognitive function.

In this study, the criteria for cognitive impairment were adjusted based on participants’ educational levels: a score of ≤17 for illiterate individuals, ≤20 for those with primary education, and ≤ 24 for those with secondary education or higher was classified as cognitive impairment, while scores above these thresholds were considered indicative of normal cognitive function (27).

### 2.6 Statistical methods

#### 2.6.1 Statistical description

Continuous measures were summarized as median (IQR) for all samples. Categorical measures were reported as frequency and percentage for crude and matched samples and only for the weighted samples.

#### 2.6.2 Control for confounding factors

To minimize the effects of confounding factors, this analysis utilized a doubly robust estimation approach, combining overlap weighting and outcome regression, to compare outcomes between the non-chronic disease with depression group and the chronic disease with depression group. Overlap weighting, a propensity score-based method, was employed to replicate essential features of randomized controlled trials. This method assigns weights to each sample proportional to the probability of belonging to the opposite treatment group, ensuring the inclusion of all available samples and achieving exact mean balance for all covariates included in the model. Compared with other weighting methods, overlap weighting has demonstrated superior precision in simulation studies.

Directed Acyclic Graphs (DAGs) are a tool used to represent and analyze causal relationships between variables, which is particularly important in causal inference. Through DAGs, researchers can identify and control for confounding factors. Confounders are variables that affect both the exposure and the outcome, potentially leading to erroneous causal inferences. By visualizing causal paths, DAGs help researchers intuitively identify which variables are confounders and determine which variables need to be controlled for in the analysis, thereby reducing bias and improving the accuracy of causal effect estimates.

**Figure 2:**
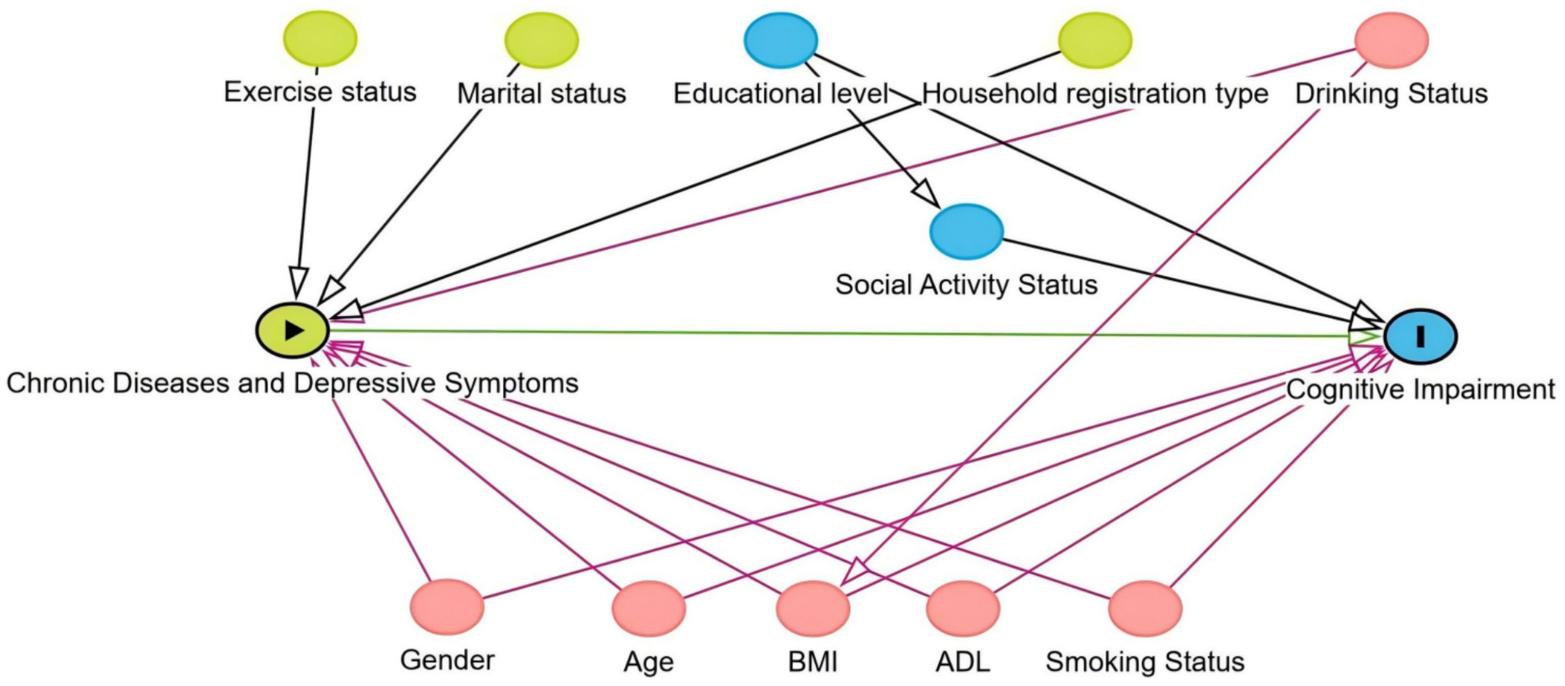
Identification of confounding factors using directed acyclic graphs (DAGs).

Initially, 11 potential confounding variables were selected based on prior literature and empirical evidence. Using DAGs (see Figure 2: https://dagitty.net/mdf5RPqYa), five key confounding factors were identified: gender (28), age (29), body mass index (BMI) (30), activities of daily living (ADL) (31), and smoking status (32). These five variables were incorporated into the overlap weighting procedure.

The newly calculated weights were then integrated into a logistic regression model, along with the identified confounding factors from overlap weighting and additional potential confounding variables, including marital status, household registration type, exercise status, education level, drinking status, and social activity status. This logistic regression model was used to adjust for confounding effects and evaluate the impact of chronic disease with depression on the incidence of cognitive impairment.

Furthermore, subgroup analyses were conducted to explore the effect of chronic disease with depression on cognitive impairment across different subgroups. Stratified analyses were performed based on gender, age (<60 years vs. ≥60 years) and education level (elementary school or below, middle school, and high school or above).

#### 2.6.3 Missing variables and data handling

The dataset exhibited missing values, particularly for marital status, household registration type, and education level, with approximately 20% of the data missing for these variables. Other variables had relatively fewer missing values (for detailed information on missing data, see Table 1). Multiple imputation (33) methods were used to handle the missing data for indicators and other variables. Five imputed datasets were constructed based on the data in Table 1, and the most appropriate imputation method was selected based on the data type. For continuous data, the Predictive Mean Matching (PMM) method was employed for imputation, while a regression imputation model was used for categorical variables to ensure data integrity. The Rubin formula was applied to obtain model estimates and compare the corrected standard errors of the imputations (34).

**Table 1:** Weighted results of baseline characteristics for all samples.

	Unweighted			Weighted^f^		
	Non-chronic diseases and depression	Chronic diseases and depression	Standardized mean difference^e^	Non-chronic diseases and depression^h^	Chronic diseases and depression^h^	Standardized mean difference^e^
No. of samples	14,015	2,875				
Demographics						
Gender, No. (%) or %						
Male	6,973 (49.75)	1,028 (35.76)	0.28	38.10	38.10	<0.01
Female	7,042 (50.25)	1847 (64.24)		61.90	61.90	
Age, median (IQR)	60.00 (46.00 to 74.00)	63.00 (49.00 to 77.00)	0.23	62.43 (52.84 to 72.02)	62.43 (53.50 to 71.36)	<0.01
BMI, median (IQR)^a^^,^^g^	23.79 (18.94 to 28.64)	24.02 (19.03 to 29.01)	0.07	24.29 (20.14 to 28.44)	24.29 (20.09 to 28.49)	<0.01
Household registration type, No. (%) or %^g^						
Non-Rural	3,727 (26.60)	584 (20.33)	0.15	25.70	20.50	0.12
Rural	10,285 (73.40)	2,289 (79.67)		74.30	79.50	
Education level, No. (%) or %						
No formal education	2,621 (18.70)	794 (27.62)	0.32	24.1	26.3	0.16
Did not complete primary school	2,862 (20.42)	702 (24.42)		21.5	24.4	
Completed private schooling	17 (0.12)	1 (0.03)		0.2	0.0	
Primary school	3,164 (22.58)	628 (21.84)		21.7	22.1	
Middle school	3,365 (24.01)	522 (18.16)		20.7	18.9	
High school	1,296 (9.25)	161 (5.60)		7.8	5.9	
Vocational school	353 (2.52)	33 (1.15)		2.2	1.2	
Associate degree	221 (1.58)	19 (0.66)		1.3	0.7	
Bachelor’s degree	105 (0.75)	14 (0.49)		0.6	0.5	
Master’s degree	10 (0.07)	0 (0)		0.0	0.0	
Doctorate degree	1 (0.01)	1 (0.03)		0.0	0.0	
Marital status, No. (%) or %^g^						
Married	12,151 (86.70)	2,332 (81.10)	0.15	83.4	81.9	0.05
Unmarried	42 (0.30)	13 (0.40)		0.3	0.4	
Divorced/Widowed	1780 (12.70)	515 (17.90)		15.9	17.1	
Other	42 (0.30)	15 (0.50)		0.4	0.6	
Smoking status, No. (%) or %^b^						
Yes	3,864 (27.57)	541 (18.82)	0.21	20.20	20.20	<0.01
No	10,151 (72.43)	2,334 (81.18)		79.80	79.80	
Drinking status, No. (%) or %						
Yes	5,424 (38.70)	794 (27.62)	0.24	32.70	28.70	0.08
No	8,591 (61.30)	2081 (72.38)		67.30	71.30	
Other factors						
Exercise status, No. (%) or %						
Yes	12,715 (90.72)	2,565 (89.22)	0.05	89.10	90.00	0.03
No	1,300 (9.28)	310 (10.78)		10.90	Doi:10.00	
Social activity status^c^						
Yes	14,015 (100)	2,875 (100)	0	100	100	0
No	0 (0)	0 (0)		0	0	
ADL, No. (%) or %^d^						
Intact	11,639 (83.05)	1,210 (42.09)	0.57	35.30	35.30	<0.01
Impaired	2,376 (16.95)	1,665 (57.91)		64.70	64.70	

a BMI: body mass index, calculated as weight in kilograms divided by height in meters squared. Prior experience and previous studies have consistently identified this variable as a confounding factor. However, as this variable was not available in the 2020 dataset, we supplemented it using data from the 2018 wave. It was subsequently treated as a primary confounding factor and handled with rigorous methods to ensure the validity of the analysis.

b Smoking status: Assign the value “Yes” to samples where “Still Smoke or already Quit” is “Yes” or where “Ever Smoked” is “Yes” and “Still Smoke or already Quit” is NA. Classify all others as “No”.

c Social Activity Status: In the 2020 (Wave 5) data, a total of 12 activities were mentioned. If a sample can perform any one of these activities Normally, their activity status is considered “Yes”.

d ADL: Activities of Daily Living; select the six items: dressing, bathing, eating, getting in and out of bed, toileting, and continence. If any of these six items has a score > 1 (Cannot be completed independently), it is recorded as “Impaired” (45).

e Absolute value of the between-group difference in means or proportions (Exposure group and control group) divided by the pooled SMD.

f Overlap weighting provided precise balance for these confounding variables.

g There were missing data for this variable, which were handled using multiple imputation. For marital status, missing data were observed in 4,894 samples (28.90%); for BMI, missing data were observed in 4,437 samples (26.20%); and for household registration type, missing data were observed in 5 samples (less than 0.01%).

h After overlap weighting, a single sample No longer represents a single data entity and thus raw counts are Not reported after overlap weighting.

#### 2.6.4 Sensitivity analysis

Propensity Score Matching (PSM) was used for sensitivity analysis, employing nearest neighbor matching with a caliper value of 0.2 to further validate the effect size obtained from the logistic regression model. Additionally, to evaluate the robustness of the observed association between chronic disease with depression and the incidence of cognitive impairment against potential unmeasured confounding factors, the E-value was calculated following the method proposed by VanderWeele and Ding (35).

A two-sided significance level of 0.05 was considered statistically significant. Odds ratios (OR) with 95% confidence intervals (CIs) were reported. Due to the exploratory nature of the study and the potential for type I errors caused by multiple comparisons, all findings should be interpreted cautiously. All statistical analyses were performed using RStudio version 4.3.3.

## 3 Results

### 3.1 General characteristics

A total of 16,890 participants were included in this study (8,889 (52.60%) were female. The median age of the participants was 61 years (IQR: 57 to 75 years), and the median body mass index (BMI) was 23.84 (IQR: 18.93 to 28.75). Data for Activities of Daily Living (ADL) were complete for all participants. Regarding smoking status, 4,423 participants (25.80%) were current smokers). Among the participants, 2,875 were identified as having chronic diseases and depression, and of these, 2,633 (91.50%) were found to have cognitive impairment.

**Figure 3:**
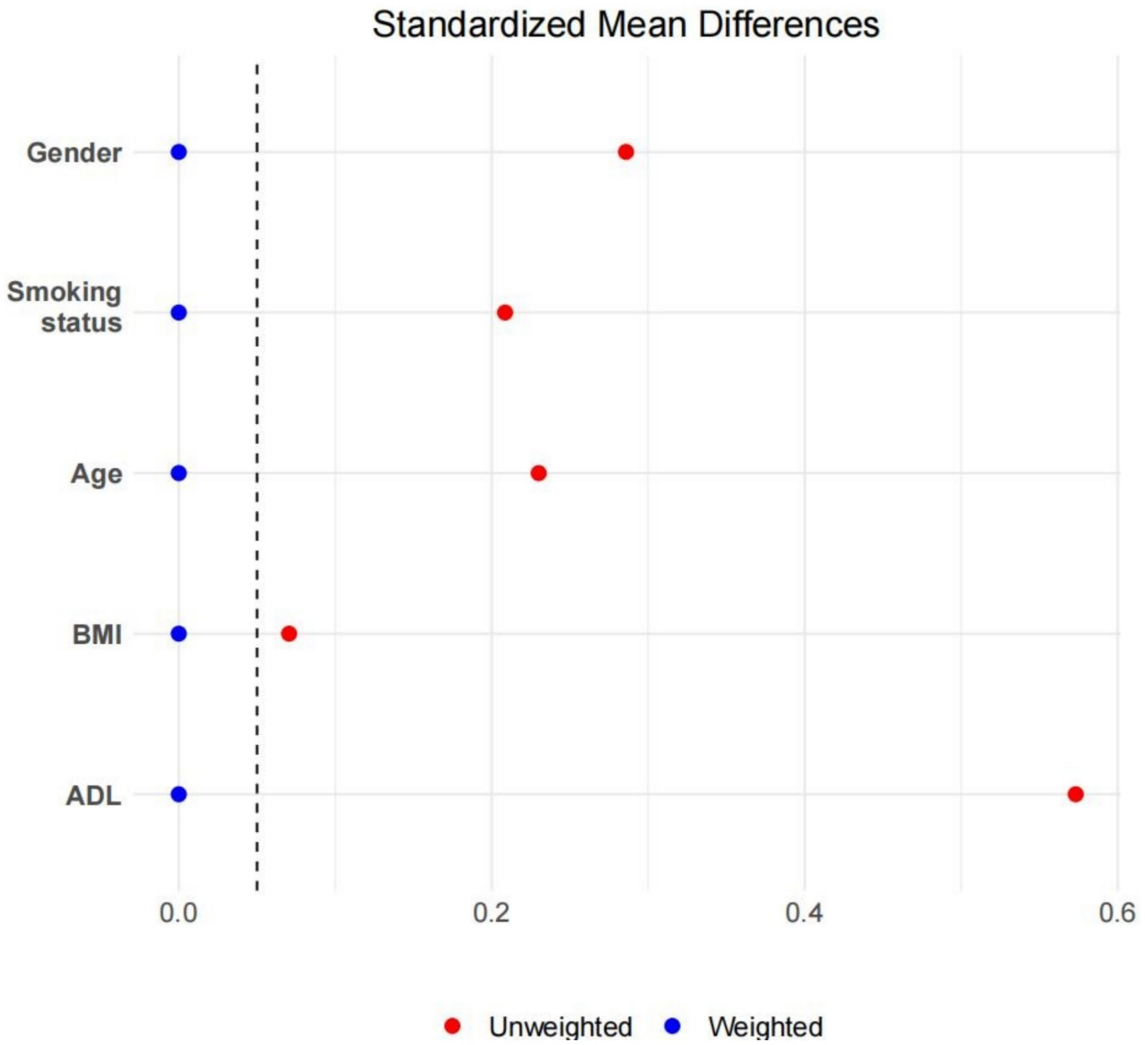
The SMD values before and after overlapping weight adjustments for confounding factors.

**Figure 4:**
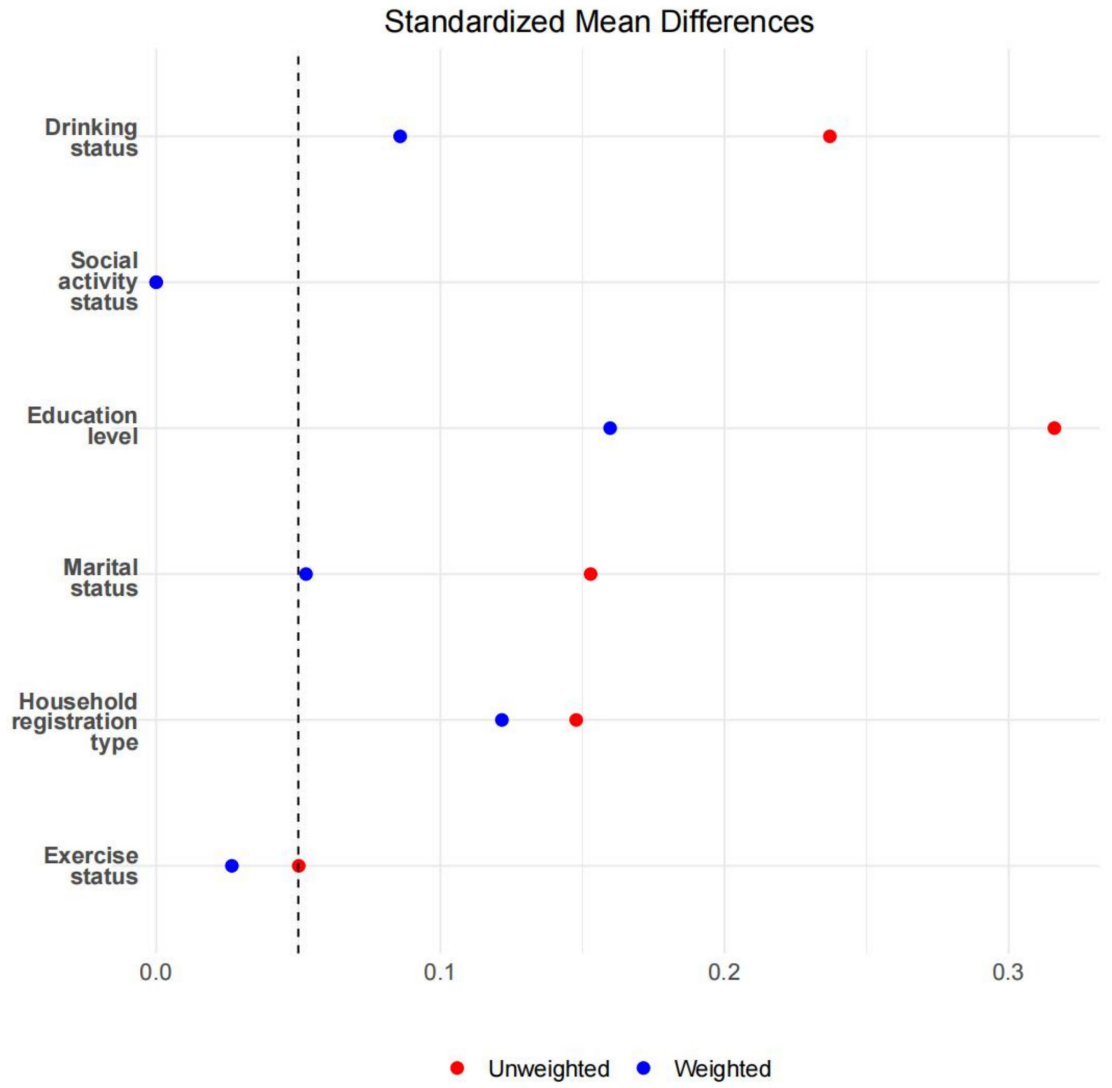
The SMD values before and after overlapping weight adjustments for potential confounding factors.

After applying overlap weighting to the five variables—gender, age, BMI, ADL, and smoking status—the maximum balance between groups was achieved at baseline (see Table 1, Figures 3, 4). Significant changes were observed in the Standardized Mean Differences (SMD) values of the confounding factors between groups. In the sample after overlap weighting, 61.90% of participants were female, the median age was 62.43 years (IQR: 52.84–72.02), the median BMI was 23.97 (IQR: 18.97–28.97), and 20.12% of participants were smokers.

### 3.2 Multivariate logistic analysis

A logistic regression was conducted on all variables after overlap weighting to explore the impact of chronic diseases and depression on the incidence of cognitive impairment. The results showed that the odds ratio (OR) was 1.22, with a 95% CI of 1.14–1.31 and *p* < 0.05, controlling for gender, age, BMI, ADL, smoking status, marital status, household registration type, exercise status, education level, drinking status, and social activity status. Individuals with chronic diseases and depression had approximately a 22.00% increased risk of developing cognitive impairment.

### 3.3 Sensitivity analysis

Logistic regression was performed on the original (unweighted) samples using propensity score matching (PSM) to validate the findings. The results indicated an OR of 1.21 (95% CI: 1.11–1.33, *p* < 0.05), further supporting the results obtained from overlap weighting. Additionally, to assess the robustness of the identified relationship between chronic diseases and depression and outcomes concerning potential unmeasured confounders, VanderWeele and Ding’s method was used to calculate the E-value, yielding an E-value of 1.44. To render the observed risk ratio (OR = 1.22) no longer significant, unmeasured confounders must be associated with chronic diseases and depression and cognitive impairment at least 1.44 times the risk. The robustness of the OR obtained from the Logistic regression after overlap weighting is moderate.

### 3.4 Subgroup analysis

Subgroup analyses were performed based on overlap weighting to examine the association in specific demographic and educational subgroups.

#### 3.4.1 Gender subgroup

In the gender-based analysis, the effect of chronic disease with depression on cognitive impairment was slightly stronger in males than in females. For males, the odds ratio (OR) was 1.13 (95% CI: 1.02, 1.26, *p* < 0.05), while for females, the OR was 1.08 (95% CI: 0.99, 1.17, *p* > 0.05).

#### 3.4.2 Age subgroup

Participants were stratified into two age groups: those younger than 60 years and those 60 years or older. The results showed that the effect size was slightly higher in the older age group. For participants aged <60 years, the OR was 1.07 (95% CI: 0.97, 1.18, *p* > 0.05), while for those aged ≥60 years, the OR was 1.12 (95% CI: 1.02, 1.22, *p* < 0.05).

#### 3.4.3 Education level subgroup

Participants were grouped into three categories based on their education level: elementary school and below, middle school, and high school or above. For the group with elementary school education or below, the OR was 1.20 (95% CI: 1.13, 1.29, *p* < 0.05). For the middle school group, the OR was 0.43 (95% CI: 0.42, 0.45, *p* < 0.05). For the high school and above group, the OR was 0.40 (95% CI: 0.38, 0.43, *p* < 0.05). The analysis revealed that as the education level increased, the risk of cognitive impairment significantly decreased.

## 4 Discussion

This study focused on middle-aged and older adults individuals, analyzing the impact of comorbid chronic diseases and depressive symptoms on the risk of cognitive impairment. By utilizing overlap weighting, multivariate logistic regression, sensitivity analysis, and subgroup analysis, we comprehensively explored the research question. Below is a detailed discussion of the study findings.

### 4.1 Combined effect of chronic diseases and depressive symptoms on cognitive impairment

The results of this study demonstrated that individuals with comorbid chronic diseases and depressive symptoms had a significantly increased risk of cognitive impairment, with an adjusted OR of 1.22 (95% CI: 1.14–1.31, *p* < 0.05). These findings support the theoretical basis that both chronic diseases and depressive symptoms are major risk factors for cognitive impairment.

Chronic diseases may negatively affect cognitive function through mechanisms such as vascular damage, inflammatory responses, and metabolic disturbances (9). Depressive symptoms, on the other hand, can further exacerbate cognitive decline through pathways involving neurotransmitter imbalances, prolonged chronic stress, and social dysfunction (12). The coexistence of these two conditions may act synergistically at both physiological and psychological levels, accelerating the decline in cognitive function.

Compared with previous studies that primarily focused on either chronic diseases or depressive symptoms independently, this study highlights the cumulative effect of their coexistence on cognitive impairment risk. These findings provide new evidence for understanding the underlying mechanisms and inform clinical interventions targeting the dual burden of chronic diseases and depressive symptoms in cognitive health management.

### 4.2 Application of overlap weighting and robustness of results

After applying overlap weighting, the standardized mean differences (SMD) of confounding factors (gender, age, BMI, ADL, and smoking status) between groups were significantly reduced, resulting in substantial improvements in the balance of baseline characteristics. This indicates that overlap weighting effectively minimized the potential impact of confounding factors, enhancing the reliability of the study results.

The robustness of the findings was further validated through sensitivity analysis. The results from logistic regression using unweighted data were consistent with those obtained after overlap weighting. Additionally, the E-value, calculated using the method proposed by VanderWeele and Ding, was 1.44. This implies that for the observed association to lose statistical significance, unmeasured confounding factors would need to be associated with both the exposure and the outcome by a risk ratio of at least 1.44 independently.

These findings demonstrate that the conclusions of this study possess a moderate degree of robustness, further supporting the reliability of the observed associations.

### 4.3 Gender subgroup analysis

In the gender subgroup analysis, the results indicated that the impact of comorbid chronic diseases and depression on cognitive impairment was statistically significant in males (OR = 1.13, 95% CI: 1.02–1.26, *p* < 0.05), but not in females (OR = 1.08, 95% CI: 0.99–1.17, *p* > 0.05). This discrepancy may reflect gender-specific mechanisms in the influence of chronic diseases and depressive symptoms on cognitive function. Men may be more vulnerable to the negative effects of chronic diseases and depression due to factors such as insufficient social support, higher levels of psychological stress, and a lower likelihood of seeking help for emotional issues. In contrast, women may benefit from higher levels of social support and stronger psychological coping abilities, which could exert a protective effect. These findings are consistent with the conclusions of previous studies (36, 37).

However, since women constitute a larger proportion (64.20%) of the total sample with comorbid chronic diseases, depression, and cognitive impairment, future research should further explore the complex role of gender in this mechanism. Specifically, whether there are unexplored biological or psychosocial factors in women that may influence their response to chronic diseases and depression. Additionally, gender differences may reflect the influence of sociocultural factors on health, suggesting that future studies could explore how gender roles affect chronic disease management and mental health across different cultural contexts.

### 4.4 Age subgroup analysis

The age subgroup analysis showed that the impact of comorbid chronic diseases and depression on cognitive impairment was more pronounced in the ≥60 years age group (OR = 1.12, 95% CI: 1.02–1.22, *p* < 0.05), while the results for the <60 years age group did not reach statistical significance (OR = 1.07, 95% CI: 0.97–1.18, *p* > 0.05). This difference may be related to the cumulative physiological and psychological effects of chronic diseases and depression. As individuals age, the burden of chronic diseases and depressive symptoms may become more significant, leading to more pronounced cognitive decline. Furthermore, older adults generally have lower cognitive reserves and reduced adaptability to chronic diseases and depressive symptoms, making them more susceptible to these factors.

These findings are consistent with previous studies (38, 39), which noted that with advancing age, cognitive abilities in the older adults gradually deteriorate, and the impact of chronic diseases and depression becomes more significant. Cognitive risk in older adults is influenced not only by physiological factors but also by social support, mental health, lifestyle, and other factors. Therefore, it is crucial to prioritize cognitive health in public health policies and clinical interventions for this high-risk group. Early screening, health interventions, and social support programs are essential to mitigate the impact of chronic diseases and depression on cognitive function.

In light of this, future research should further investigate the mechanisms of age-related cognitive decline, particularly focusing on how lifestyle improvements and enhanced mental health interventions can strengthen cognitive reserve in older adults and delay cognitive deterioration. Integrating such interventions could play a critical role in mitigating cognitive decline, offering valuable insights for enhancing cognitive health in aging populations.

### 4.5 Education level subgroup analysis

The education level subgroup analysis demonstrated that the impact of comorbid chronic diseases and depression on the risk of cognitive impairment significantly decreased with higher levels of education. In the elementary school or below group, the odds ratio (OR) was 1.20 (95% CI: 1.13–1.29, *p* < 0.05), whereas in the high school or above group, the OR was only 0.40 (95% CI: 0.38–0.43, *p* < 0.05). These findings align with the Cognitive Reserve Hypothesis, which suggests that higher education enhances cognitive reserve, enabling individuals to better withstand pathological damage without exhibiting clinical symptoms.

#### 4.5.1 Cognitive reserve

Individuals with higher education are believed to have greater cognitive reserve, allowing them to tolerate brain pathology, such as vascular damage or neurodegeneration, without manifesting cognitive decline (40, 41). This reserve serves as a buffer, mitigating the effects of chronic diseases and depressive symptoms on cognitive function.

#### 4.5.2 Enhanced problem-solving and coping skills

Higher education equips individuals with better problem-solving abilities and adaptive coping mechanisms, enabling them to manage chronic diseases and psychological stress more effectively (42). These skills may help reduce the risk of cognitive impairment.

Health Literacy: Educational attainment is closely linked to improved health literacy, which facilitates better management of chronic diseases, adherence to medical treatments, and adoption of healthier lifestyles, all of which contribute to maintaining cognitive health (43).

#### 4.5.3 Socioeconomic and lifestyle factors

Individuals with higher education levels are more likely to have better access to healthcare, healthier diets, more opportunities for physical activity, and greater social engagement. These factors are well-documented as protective against cognitive decline (44).

These findings underscore the importance of improving education levels and encouraging participation in cognitive activities as effective strategies for preventing cognitive impairment. Public health interventions aimed at enhancing education and promoting lifelong learning could play a critical role in reducing the burden of cognitive decline in the population.

### 4.6 Clinical and public health implications

This study highlights the significant impact of chronic diseases and depressive symptoms on the risk of cognitive impairment in middle-aged and older adults populations, emphasizing the importance of early and comprehensive management of both chronic diseases and depressive symptoms. In clinical practice, personalized intervention strategies should be developed for high-risk populations, such as older males and individuals with lower education levels. Preventive measures should include controlling chronic diseases, alleviating depressive symptoms, and enhancing cognitive reserve to prevent or delay the onset of cognitive impairment.

Furthermore, this study provides data to support the formulation of public health policies. It underscores the need to strengthen mental health services and cognitive health management in middle-aged and older adults populations, promoting targeted initiatives to address the dual burden of chronic diseases and mental health issues in this vulnerable group. Given the findings of this study, we suggest that policymakers focus on improving health management for middle-aged and older adults populations, particularly those with high-risk factors such as older age and lower education levels. Policies should encourage regular health screenings, especially for chronic diseases and depressive symptoms, and incorporate interventions that address both physical and mental health to reduce the risk of cognitive decline.

Additionally, the study reveals significant differences in the impact of chronic diseases and depressive symptoms on cognitive impairment based on gender, age, and education level. Therefore, policymakers should consider designing health interventions tailored to these subgroups. For example, interventions targeting men should focus more on the identification and treatment of depressive symptoms, while those targeting individuals over the age of 60 should include cognitive health protection measures. Moreover, populations with lower educational attainment should receive additional health education and psychological support services to mitigate the impact of chronic diseases and mental health issues on cognitive health.

### 4.7 Study limitations and future directions

Despite the reliability of the findings validated through overlap weighting and sensitivity analyses, this study has several limitations.

First, the ability to infer causality is limited, as this study design does not allow for definitive conclusions about the long-term effects of comorbid chronic diseases and depressive symptoms on cognitive impairment. Future longitudinal studies are needed to further investigate these relationships over time. Second, although the E-value was used to assess the potential impact of unmeasured confounding factors, residual confounding cannot be entirely ruled out. This highlights the need for caution in interpreting the results and the importance of considering additional confounders in future studies. Finally, the sample source and context may limit the external validity of the findings. The study conclusions should be further validated in diverse populations and regions to ensure generalizability.

Future research should focus on exploring the mechanisms underlying the interaction between chronic diseases, depressive symptoms, and cognitive impairment, as well as testing the effectiveness of targeted interventions in reducing cognitive decline across different demographic and clinical settings.

## 5 Conclusion

This study systematically analyzed the impact of comorbid chronic diseases and depressive symptoms on the risk of cognitive impairment in middle-aged and older adults populations. The results demonstrated that the combined effect of these conditions significantly increased the risk of cognitive impairment, with notable variations observed across specific subgroups. These findings not only enhance our understanding of the underlying mechanisms but also provide a scientific basis for the prevention and intervention of cognitive impairment. Future research should further explore the mechanisms of interaction between chronic diseases and depressive symptoms and validate these findings in diverse populations to ensure broader applicability and generalizability.

## Data Availability

The datasets presented in this study can be found in online repositories. The names of the repository/repositories and accession number (s) can be found below: https://charls.charlsdata.com/.
